# Ala344Pro mutation in the *FGFR2* gene and related clinical findings in one Chinese family with Crouzon syndrome

**Published:** 2012-05-15

**Authors:** Ying Lin, Siming Ai, Chuan Chen, Xialin Liu, Lixia Luo, Shaobi Ye, Xuanwei Liang, Yi Zhu, Huasheng Yang, Yizhi Liu

**Affiliations:** 1 State Key Laboratory of Ophthalmology, Zhongshan Ophthalmic Center, Sun Yat-sen University, Guangzhou, China; 2 Department of Pharmacology (State-Province Key Laboratories of Biomedicine-Pharmaceutics of China), Harbin Medical University, Harbin, Heilongjiang, China

## Abstract

**Purpose:**

The purpose of this study was to investigate the fibroblast growth factor receptor 2 (*FGFR2*) gene in three Chinese patients with Crouzon syndrome and to characterize the related clinical features.

**Methods:**

A single family underwent complete ophthalmic examinations, and three patients were diagnosed with Crouzon syndrome. Genomic DNA was extracted from leukocytes of peripheral blood collected from members of the family as well as from 100 unrelated control subjects from the same population. Exons 8 and 10 of *FGFR 2* were amplified by polymerase chain reaction (PCR) and directly sequenced. We performed ophthalmic examinations, including best-corrected visual acuity, slit-lamp examination, fundus examination, Pentacam, Goldmann perimetry, and computed tomography (CT) of the skull.

**Results:**

The three patients were affected with shallow orbits and ocular proptosis, accompanied by mid-face hypoplasia and craniosynostosis, but had clinically normal hands and feet. A heterozygous *FGFR2* missense mutation c.1030G>C (Ala344Pro) in exon 10 was identified in the affected individuals, but not in any of the unaffected family members or the normal controls. The mutation we identified has not previously been reported, either in China or abroad.

**Conclusions:**

Although *FGFR2* mutations and polymorphisms have been reported in various ethnic groups, especially in the area of osteology, we report, for the first time, the identification of one new *FGFR2* gene mutation in Chinese patients with Crouzon syndrome.

## Introduction

Crouzon syndrome (CS), characterized by craniosynostosis, shallow orbits, ocular proptosis, mid-face hypoplasia, and a curved, beaklike nose, is an autosomal-dominant, inherited disorder of the most common craniosynostosis syndrome [1-6].

Until now, it was known that craniosynostosis bears a strong link to the fibroblast growth factor receptors (FGFRs). FGFRs are trans-membrane proteins and their ligand-binding specificity depends on the third extra-cellular Ig-like domain, which is subject to alternative splicing that generates a variety of receptor iso-forms. Three different splice variants, IgIIIa, IgIIIb, and IgIIIc, have been identified [7-10].

It is known that Crouzon syndrome is usually caused by mutations in the fibroblast growth factor receptor 2 (*FGFR2*) gene, located on chromosome 10q26 [11,12]. Most mutations have been described in *FGFR2* and result in Apert, Crouzon, Jackson-Weiss, or Pfeiffer syndromes. Over 50 different mutations have been described in Crouzon syndrome, with approximately 95% of the cases having mutations in just two exons of the gene, IIIa (8) and IIIc (10), which encode the extra-cellular immunoglobulin-like III (IgIII) domain of the protein [7].

This study reported the mutational analysis of one Chinese family with Crouzon syndrome at the gene level, along with related clinical features, and identified one heterozygous mutation.

## Methods

### The Crouzon syndrome family

**Figure 1:**
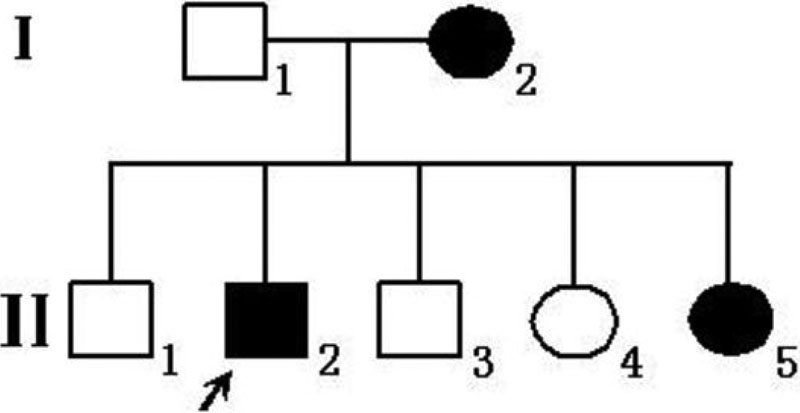
The pedigree of a Chinese family with Crouzon syndrome. Squares denote males and circles denote females. The shaded symbols indicate ophthalmologist-confirmed Crouzon syndrome. The arrow points to the proband.

Three patients in one Chinese family (Figure 1) were diagnosed, through the Zhongshan Ophthalmic Center, as having Crouzon syndrome. We performed ophthalmic examinations, as follows: Visual acuity was examined using the Early Treatment Diabetic Retinopathy Study (ETDRS) chart (Precision Vision, La Salle, IL). An anterior segment photograph was obtained using a BX 900 Slit Lamp (Haag-Streit, Bern, Switzerland). Anterior segment measurements were taken with Pentacam HR version 70700 (Oculus, Wetzlar, Germany). In addition, computed tomography (CT) and physical examinations were performed to exclude systemic diseases.

### Sample collection

The affected family was identified at the Zhongshan Ophthalmic Center. One hundred subjects who had no diagnostic features of Crouzon syndrome were recruited from the same population to serve as normal controls. After informed consent was obtained from all participating individuals, according to the principles of the Declaration of Helsinki, venous blood samples were collected for genomic DNA extraction from peripheral blood leucocytes, using standard protocols.

### Mutation detection

Exons 8 and 10 of *FGFR2* were amplified by polymerase chain reaction (PCR) with primers (Table 1) [13]. Briefly, PCR was conducted in 50-μl reactions. The cycling profile included one cycle at 94 °C for 5 min, followed by 40 cycles at 94 °C for 45 s, 52–66 °C for 45 s, and 72 °C for 45 s, as well as one cycle at 72 °C for 10 min. The PCR products were sequenced from both directions with an ABI3730 Automated Sequencer (PE Biosystems, Foster City, CA). The sequencing results were analyzed using Chromas (version 2.3; Technelysium Pty Ltd, Brisbane, QLD, Australia); they were compared with the reference sequences in the database at the National Center for Biotechnology Information (NCBI; NC_000010.10).

**Table 1:** Primers used for PCR.

**Exon**	**Forward (5′-3′)**	**Reverse (5′-3′)**	**Product size (bp)**	**Annealing temperature (°C)**
FGFR2–8 (IIIa)	GGTCTCTCATTCTCCCATCCC	CCAACAGGAAATCAAAGAACC	325	61
FGFR2–10 (IIIc)	CCTCCACAATCATTCCTGTGTC	ATAGCAGTCAACCAAGAAAAGGG	257	61

Summary of the primers and products length used for the amplification of the exons of *FGFR2*.

## Results

### Clinical data

**Figure 2:**
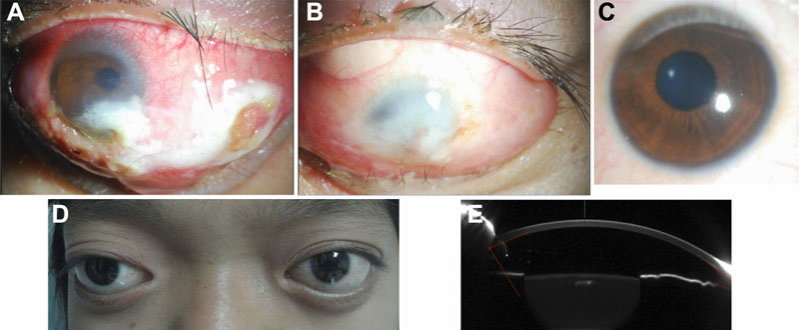
Examination results of Patients II-2 and II-4. **A**: The anterior segment photograph of the right eye of Patient II-2. The cornea had serious exposure keratitis. **B**: The anterior segment photograph of the left eye of Patient II-2. The cornea was opaque. **C**: The anterior segment picture of Patient II-4. **D**: Ocular proptosis of Patient II-4. **E**: The anterior segment picture of Patient II-4 by Pentacam.

The Chinese family studied in this report was from the southern area of China. Three individuals, in two successive generations, were found to have the same congenital disease (Figure 1). These patients had shallow orbits and ocular proptosis, accompanied by mid-face hypoplasia, craniosynostosis, a curved, beaklike nose, but clinically normal hands and feet. Although they had had normal vision since early childhood, just displaying a surprised look, their vision was getting worse and worse as they aged (Figure 2A,B).

The I-2 patient (52 years old; female) had already had eyeball extraction surgery because of serious exposure keratitis, corneal scarring and serious eye pain. As a result, we don’t have the pictures and examination results for her.

The visual acuity of the II-2 patient (22 years old; male), as measured by logarithm of the minimum angle of resolution(Log MAR), was LP (light perception, OD) and NLP (no light perception, OS). The cornea of the right eye had very serious exposure keratitis (Figure 2A) and the left eye had chronic corneal scarring (Figure 2B), with no light perception. No abnormalities were detected in the lens, retina, choroid, or optic nerve of the left eye, and we were unable to examine the lens, retina, choroid, and optic nerve of the right eye, or the axial length and the eye prominence, because of the serious corneal scar and the keratitis.

Figure 2C shows the right eye of the II-4 patient (10 years old; female). She had shallow orbits and ocular proptosis, accompanied by mid-face hypoplasia (Figure 2D) and clinically normal hands and feet. The visual acuity of the II-4 patient was 0.7 (OD) and 0.4 (OS). The refractive error of the II-2 patient was −1.75D, with −1.50 astigmatism (OD) and −1.25D, with −1.50 astigmatism (OS). Axial lengths were 24.54 mm (OD) and 24.42 mm (OS). No abnormalities were detected in the lenses, retinas, choroids, or optic nerves. The anterior segment photograph is shown in Figure 2E; the anterior chamber depths were 3.15 mm (OD) and 3.17 mm (OS). The prominence of the II-5 patient was 17 mm (OD) and 16 mm (OS).

### Mutation screening

**Figure 3:**
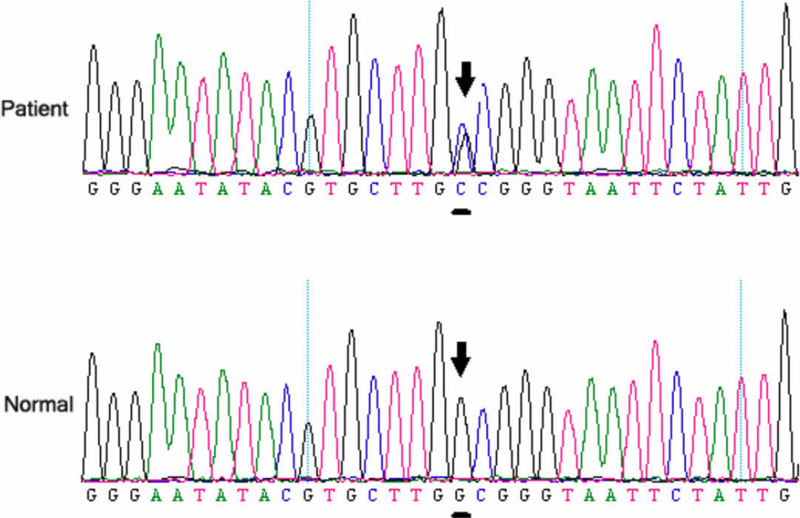
DNA sequence of a part of the *FGFR2* gene in the affected patients and unaffected individuals. A heterozygous missense mutation c.1030G>C in exon 10 was identified in the three affected individuals, but not in any of the unaffected family members or the normal controls. The mutation causes the Alanine 344 codon (GCG) to change to a Proline codon (CCG).

A heterozygous missense mutation c.1030G>C in exon 10 of *FGFR2* (Figure 3) was identified in the three affected individuals, but not in any of the unaffected family members, nor in the normal controls. The mutation causes the Alanine 344 codon (GCG) to change to a Proline codon (CCG).

## Discussion

In this study, we found one mutation in exon 10 of *FGFR2* that is associated with Crouzon Syndrome: c.1030G>C. This mutation, rather than a rare polymorphism in the normal population, is the causative mutation in the family.

The c.1030G>C mutation (Ala344Pro) was identified, for the ﬁrst time, in *FGFR2* in Chinese patients; it has not previously been reported either in China or abroad.

Previously described mutations causing craniosynostosis are widely distributed across the FGFR2 protein, yet the majority localize in some amino acids that form the S-S bond in the IgIIIa/IIIc domain, resulting in the disruption of the protein’s structure, dimerization, and activation of the receptor [14].

The ocular manifestations in the conditions caused by *FGFR* mutations included down-slanting palpebral fissures, shallow orbits and proptosis, hypertelorism, strabismus, ocular anterior chamber dysgenesis, optic nerve hypoplasia, scleralization of the cornea, and corectopia in craniosynostosis syndromes [15-17]. In addition, the syndrome gets worse and worse with aging because of the exposure keratitis, which suggests that a suitable orbital decompression surgery at a reasonable time is of vital importance, to prevent the progress of Crouzon syndrome.

Until now, Crouzon syndrome has generally been treated by Le Fort III advancement osteotomies, followed by distraction [18,19]. Quadrangular osteotomy, which is specifically designed to correct maxillary hypoplasia extending to the infra-orbital rim, has also been performed. The infra-orbital rim can be moved forward, thereby improving exorbitism, but the nasion and posterior zygomatic arch cannot be changed [18,20]. In our patient II-2, we performed a quadrangular osteotomy with midline split to simultaneously achieve anterior advancement and lateral expansion. This combination of anterior and lateral distraction may enable us to obtain a suitable orbital space.

In summary, this study identified one novel mutation of *FGFR2* in a Chinese family with Crouzon syndrome. This finding expands the mutation spectrum of *FGFR2* and is useful and valuable for genetic counseling and prenatal diagnosis in families with Crouzon syndrome without serious ocular disorders.
